# Physicochemical Properties and Cellular Responses of Strontium-Doped Gypsum Biomaterials

**DOI:** 10.1155/2012/976495

**Published:** 2012-06-07

**Authors:** Amir Pouria, Hadis Bandegani, Milad Pourbaghi-Masouleh, Saeed Hesaraki, Masoud Alizadeh

**Affiliations:** ^1^Nanotechnology and Advanced Materials Department, Materials and Energy Research Center, P.O. Box 31787/316, Karaj 3177983634, Iran; ^2^Ceramics Department, Materials and Energy Reserach Center, P.O. Box 31787/316, Karaj 3177983634, Iran

## Abstract

This paper describes some physical, structural, and biological properties of gypsum bioceramics doped with various amounts of strontium ions (0.19–2.23 wt%) and compares these properties with those of a pure gypsum as control. Strontium-doped gypsum (gypsum:Sr) was obtained by mixing calcium sulfate hemihydrate powder and solutions of strontium nitrate followed by washing the specimens with distilled water to remove residual salts. Gypsum was the only phase found in the composition of both pure and gypsum:Sr, meanwhile a shift into lower diffraction angles was observed in the X-ray diffraction patterns of doped specimens. Microstructure of all gypsum specimens consisted of many rod-like small crystals entangled to each other with more elongation and higher thickness in the case of gypsum:Sr. The Sr-doped sample exhibited higher compressive strength and lower solubility than pure gypsum. A continuous release of strontium ions was observed from the gypsum:Sr during soaking it in simulated body fluid for 14 days. Compared to pure gypsum, the osteoblasts cultured on strontium-doped samples showed better proliferation rate and higher alkaline phosphatase activity, depending on Sr concentration. These observations can predict better in vivo behavior of strontium-doped gypsum compared to pure one.

## 1. Introduction

Because strontium is chemically and physically similar to calcium, it is a trace element that accumulates in the skeleton, preferably in new trabecular bone, depending on the skeletal site. Sr content increases in the sequence diaphysis of the femur, lumbar vertebra, and iliac crest [1].

Strontium (Sr) salts were found to stimulate bone formation and inhibit bone resorption both in vitro and in vivo [2]. The oral strontium-containing drugs have recently been recommended as drug associated with treating osteoporosis [3]. The stimulatory effects of strontium on bone collagen synthesis has been reported in vitro, while neither calcium nor sodium salts were effective [4].

In the past decade, a wide range of bioceramics such as hydroxyapatite, tricalcium phosphate, octacalcium phosphate, and bioactive glasses have been studied for orthopaedic applications [5–7]. Because of the beneficial effects of strontium on the treatment of bone diseases and defects, many studies have focused on synthesis, characterization, and animal modeling of strontium-containing bioceramics. Many authors attempted to incorporate Sr into crystal lattice of calcium phosphates either through a high temperature synthesis [8] or by precipitation during a setting reaction of hydroxyapatite forming cement [9]. Strontium-containing hydroxyapatite was mixed with bone cement [10, 11] to promote osteoblast attachment and mineralization in vitro [12] and accelerate bone growth and osteointegration in vivo [13]. Strontium-substituted beta-tricalcium phosphate was synthesized and used as a reactant of calcium phosphate cement with a Sr^2+^ release range of 12–30 ppm. Sr-doped hydroxyapatite was used as plasma-sprayed coating layer where a simulated cell response was observed for this layer compared to pure hydroxyapatite [14]. Incorporation of strontium into bioactive glasses has been also reported by some authors. Abou Neel et al. [15] reported structural and physical properties of melt-derived phosphate-based glasses in which positive effect of Sr-doped glasses on viability of human osteoblastic cells was reported. Physicochemical and in vitro cellular properties of sol-gel-derived bioactive glasses based on CaO-SrO-SiO_2_-P_2_O_5_ system were reported by Hesaraki et al. [16].

Calcium sulfates are biocompatible and biodegradable materials used for the treatment of bone and periodontal defects for many years. Calcium sulfate can be used as a bulk material, space filler, and vehicle for a controlled release of certain drugs, associated with other graft materials [17–19]. A number of positive clinical experiences are available with calcium sulfate in bone substitution procedures [20]. Gypsum is dihydrate form of calcium sulfate (CaSO_4_-2H_2_O) synthetically made from Plaster of Paris, hemihydrate form of calcium sulfate (CaSO_4_-1/2H_2_O). Gypsum with a microstructure comprising a lot of small crystals entangled to each other, suggested that provides a more efficient environment for bone repair. It has been stated that gypsum not only is passive osteoconductive material but also might has a potential to be osteoinductive due to its special crystal structure, and high calcium content [21].

The aim of this study was to incorporate various contents of strontium ions into calcium sulfate dihydrate (gypsum) crystal lattice and to investigate the effect of this substitution on some physicochemical, structural and in vitro cellular properties of the material.

## 2. Materials and Methods

### 2.1. Starting Materials and Trade Marks

The following starting materials were used in this study: alpha-calcium sulfate hemihydrate (*α*-CSH, Aldrich, USA), strontium nitrate (Merck, Germany), sodium chloride (Merck, Germany), potassium chloride (Merck, Germany), calcium chloride dihydrate (Merck, Germany), sodium hydrogen carbonate (Merck, Germany), dipotasium hydrogen phosphate trihydrate (Merck, Germany), sodium sulfate decahydrate (Merck, Germany), magnesium chloride hexa-hydrate (Merck, Germany), tris-hydroxymethyl aminomethane (Merck, Germany), and hydrochloric acid (Merck, Germany). Except *α*-CSH and strontium nitrate, all the above mentioned precursors were used for the preparation of simulated body fluid (SBF) solution.

### 2.2. Preparation of Strontium-Doped Gypsum Specimens

Strontium-doped gypsum bioceramics (gypsum:Sr) were prepared from the setting process of calcium sulfate hemihydrate. Calcium sulfate hemihydrate was mixed with aqueous solution of strontium nitrate at a solid to liquid loading of 2 g/mL and the obtained paste was poured into a disc-shaped Teflon mold (10 mm in diameter and 3 mm in height) and left to set. Various concentrations of strontium nitrate were used to achieve gypsum product with different concentrations of strontium doped ions. Pure distilled water was also used to fabricate gypsum product without any additive as control sample (G-0).  Table 1 presents details of various paste formulations of Sr-doped gypsum specimens along with the code of each formulation. When the pastes were completely hardened (set), the specimens were taken from the mold, incubated in 100% humidified atmosphere at 37°C for 24 h, washed with distilled water for several times to remove the residuals and soluble salts, and,finally, dried at room temperature for 72 h.

### 2.3. Experimental Procedures

#### 2.3.1. Concentration of Strontium in Gypsum:Sr

The concentration of strontium ions in the Sr-doped gypsum specimens was measured using inductively coupled plasma-atomic emission spectroscopy (ICP-AES) technique ARL 3410.

#### 2.3.2. Phase Composition

Phase compositions of the specimens were determined using an X-ray diffractometer device (XRD, Philips PW 3710) with Cu-K*α* radiation, an operating voltage of 40 kV, and a scanning rate of 0.02 2*θ*/s. For this purpose, the same weight of each specimen in powdered form was delivered to XRD unit. The XRD patterns were assessed by X'Pert HighScore software, version 1.0d, 2003 (PANalytical B.V., Almelo, The Netherlands).

#### 2.3.3. Chemical Groups

To investigate the structural characteristics of the specimens, Fourier-transform infrared (FTIR) spectroscopy was used with KBr powder as a standard. Transparent discs were prepared by mixing the sample and the standard KBr at the KBr/sample ratio of 10 (in w/w). The FTIR spectra of the specimens were collected at 4000–400 cm^−1^ region with 2 cm^−1^ resolution using Bruker Vector 33.

#### 2.3.4. Microstructural Observations and Elemental Image Analysis

The microstructure of the pure gypsum and gypsum:Sr specimens were observed using an scanning electron microscope (TESCAN, VEGA II, XMU) operated at an accelerating voltage of 40 kV and equipped with energy dispersive X-ray analyzing (EDXA) device.

#### 2.3.5. Density

The powder density of the specimens was measured using a gas pycnometer device (Accupyc 1330, Micromeritics). For this purpose the dried hardened samples were powdered, passed through a 230 mesh sieve, and then characterized.

#### 2.3.6. Compressive Strength

To understand the effect of incorporation of Sr^2+^ ions into gypsum on its mechanical strength, gypsum:Sr cylindrical specimens (6 mm in diameter and 12 mm in height) were fabricated in Teflon mold as described in Section 2.2. Each sample was kept in a SBF solution for 24 h and the compressive strength of wet specimens was recorded using a universal testing device (Zwick/Roell-HCR 25/400) at a crosshead speed of 1 mm/min. In this study, the SBF solution with a chemical composition resembling that of human blood plasma (solution with ionic concentration of Na^+^ 142.0, K^+^ 5.5, Mg^2+^ 1.5, Ca^2+^ 2.5, Cl^−^ 147.8, HCO_3_^−^ 4.2, HPO_4_^2−^ 1.0 and SO_4_^2−^ 0.5 mM) was prepared by dissolving the chemical reagents in distilled water, buffering it with tris-hydroxymethyl aminomethane and adjusting its pH to 7.4 using hydrochloric acid [22].

#### 2.3.7. Solubility

The solubility of gypsum and gypsum:Sr specimens were investigated through immersing disc-shaped specimens in the SBF solution at solid to solution loading of 1.6 g/100 mL and measuring the concentration of Ca and Sr ions released from the specimens into the solution (using ICP-AES instrument) as a function of time. Note that after each evaluating period, the whole volume of the SBF solution was extracted for analysis and the sample was immediately fed with a fresh solution. Cumulative concentration of each ion was calculated using the following expression:

(1) $${\left[\text{Sr}\right]}_{n}=\overset{i=n}{\underset{i=1}{\sum }}{\left[\text{Sr}\right]}_{i},$$

where [Sr]_*n*_ is cumulative concentration of Sr ions at the *n*th period of evaluation and *i* is the evaluating time interval.

#### 2.3.8. Cell Proliferation and Alkaline Phosphatase Activity

To study the effect of incorporation of strontium into gypsum biomaterial on the viability and alkaline phosphatase activity of the osteoblastic cells, the in vitro tests were carried out using the human osteosarcoma (G-292) cells (NCBI C 116 National Cell Bank of Iran). The cells were cultured in tissue culture polystyrene (PS) flasks (Falcon, USA) at 37°C under 5% CO_2_ atmosphere in Dulbecco's modified Eagle's medium (DMEM) with l-glutamine, supplemented 10% fetal bovine serum (FBS) and antibiotic antimycotic (100 units penicillin G sodium, 100 mg streptomycin sulfate, and 0.25 mg amphotericin B in saline) and harvested after the treatment with 0.05% trypsin-EDTA.

The proliferation of osteoblast cells onto the gypsum specimens was measured by MTT assay. The samples were sterilized with 70% ethanol and seeded with the cells at 3 × 10^4^ cells/disc. Polystyrene discs with the surface area similar to gypsum specimens were prepared from the tissue culture plate and similarly were seeded with the cells as control specimens. The specimen/cell constructs were placed into 24-wells culture plates and left undisturbed in an incubator for 4 h to allow the cells to attach to them. Then, 3 mL of culture medium was added into each well and the cell/specimen constructs were cultured in a humidified incubator at 37°C with 95% air and 5% CO_2_ for 1, 3, and 7 days. The medium was changed every 3 days. After each period, the medium was removed and 2 mL of MTT, 3-(4,5 dimethylthiazol-2-yl)-2,5-diphenyl tetrazolium bromide, solution was added to each well. Following incubation at 37°C for 4 h in a fully humidified atmosphere of 5% CO_2_/95% air, MTT was taken up by active cells and reduced in the mitochondria to insoluble purple formazan granules. Subsequently, the medium was discarded and the precipitated formazan was dissolved in dimethylsulfoxide, DMSO, (150 mL/well), and optical density of the solution was read using a microplate spectrophotometer (BIO-TEK Elx 800, Highland park, USA) at a wavelength of 570 nm. The optical density (OD) was measured at the wavelength of 590 nm using a multiwell microplate reader (ICN, Switzerland).

The osteoblast activity was determined by measuring the level of alkaline phosphatase (ALP) produced by G-292 cells. The cells were seeded on the samples under the same culturing condition described above and the level of ALP activity were determined on days 1, 3, and 7. The G-292 cell lysates were frozen and thawed three times to disrupt the cell membranes. ALP activity was determined at 405 nm using p-nitrophenyl phosphate in diethanolamide buffer as chromogenic substrate.

#### 2.3.9. Statistical Analysis

Data were processed using Microsoft Excel 2003 software and the results were produced as mean ± standard deviation of at least 4 experiments. Significance between the mean values was calculated using standard software program (SPSS GmbH, Munich, Germany) and the *P* ≤ 0.05 was considered significant.

## 3. Results and Discussion

### 3.1. Concentration of Strontium in Sr-Doped Gypsum

In this study Sr-doped gypsum was prepared through conversion of calcium sulfate hemihydrate phase into calcium sulfate dihydrate through a hydraulic reaction at the presence of Sr solution. The mechanisms of gypsum formation have been discussed elsewhere [23]. The samples were kept in a 100% humid atmosphere until all starting hemihydrate phase is converted to dihydrate form (gypsum). The samples were washed with distilled water to avoid the presence of residual soluble strontium or calcium nitrates in the formed porous matrix.  Table 2 presents the concentration of Ca and Sr elements in gypsum and gypsum:Sr measured by ICP technique. The Sr content increased with increasing the amount of added Sr salt. The molar concentration of Sr in the gypsum:Sr specimens is in the range of 0.4–5.2 mol% of Ca. The normal concentration of Sr in human skeleton is ~3.5 mol % of Ca [24]. The results can describe why the strontium was not added in more concentrations than the selected values.

### 3.2. Phase Composition

**Figure 1:**
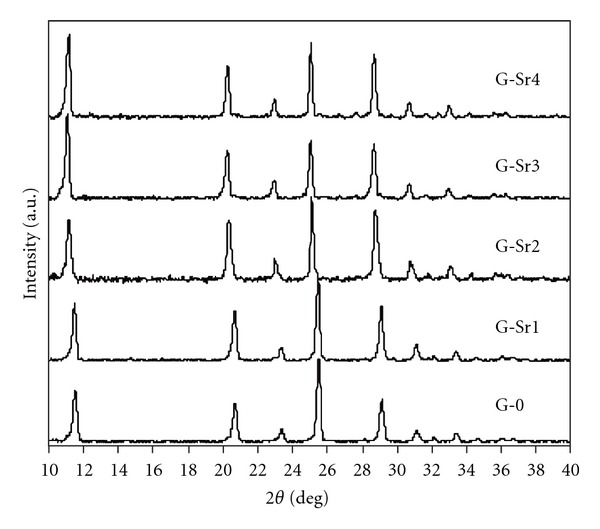
The XRD patterns of gypsum specimens with content of doped Sr.

Figure 1 shows the XRD patterns of the calcium sulfate specimens with various contents of Sr dopant after hardening and washing with distilled water. All specimens show sharp and narrow peaks of gypsum and no other phases are found in the patterns. The X-ray diffraction patterns of gypsum:Sr specimens reveal a shift to lower diffraction angles when Sr concentration is increased, indicating that Sr ions are incorporated into lattice structure of gypsum crystals. In comparison with pure gypsum, the X-ray diffraction patterns of Sr-doped gypsum specimens also show an increase in the intensity of the peaks at 2*θ* = 11.8° corresponding to 020 crystal planes which is indicative of a preferred direction of gypsum crystal growth during hydration process.

### 3.3. Chemical Groups

**Figure 2:**
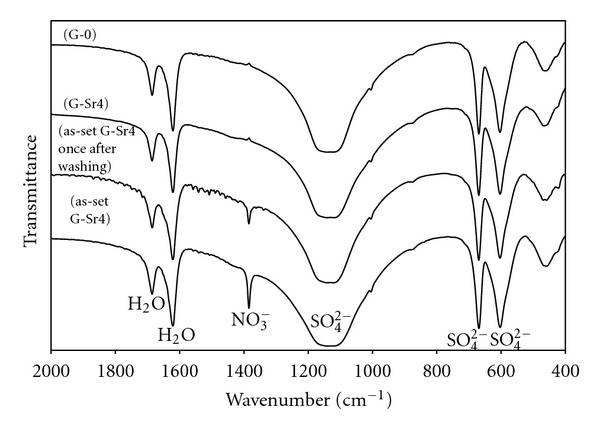
The FTIR spectra of pure gypsum (G-0) and Sr-doped gypsum (G-Sr4) in comparison with as-set G-Sr4 and G-Sr-4 specimen once after washing procedure.

The FTIR spectra of pure gypsum and Sr-doped gypsum (G-Sr4) are shown in Figure 2. The spectrum of as-set G-Sr4 specimen (hardened specimen before washing with distilled water) and its spectrum once after washing with distilled water are also shown for comparison. The FTIR spectra show the stretching bands associated to the functional groups of the gypsum components, that is, H_2_O and SO_4_^2−^ groups labeled in the corresponding Figures. The FTIR spectra of all other Sr-doped gypsum specimens were closely similar to that of G-Sr4 and thus have not been illustrated. Decreased intensity of nitrate band once after washing and disappearance of this band after completion of the washing procedure confirms removal of the residual additive. As it is observed, the spectrum of gypsum:Sr is similar to the spectrum of pure gypsum and no extra bands are found.

### 3.4. Microstructural Observations and Elemental Image Analysis

**Figure 3:**
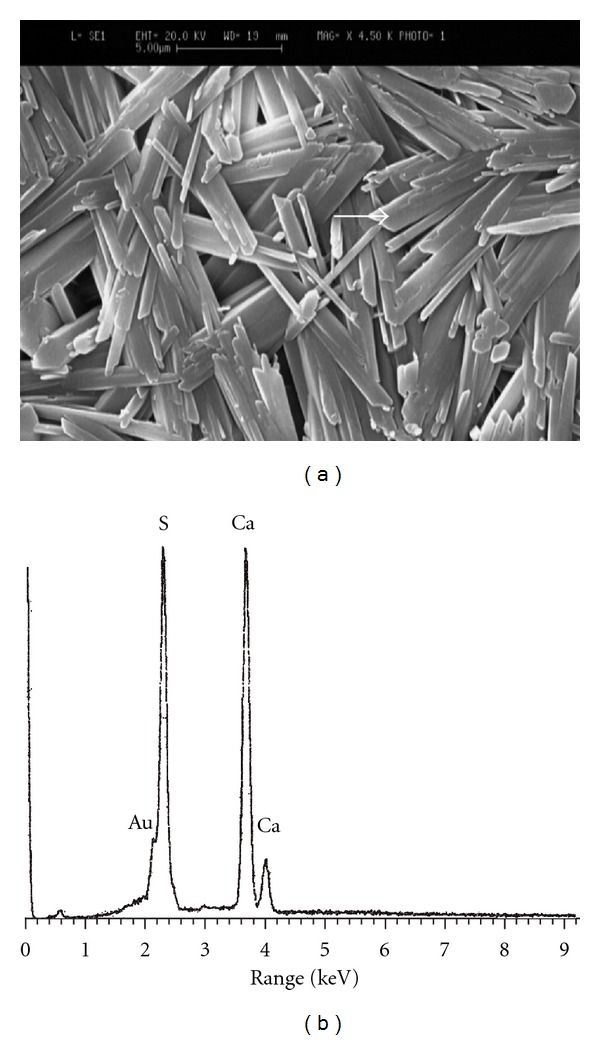
The SEM image of pure gypsum (a) along with its corresponding EDXA patterns (b).

**Figure 4:**
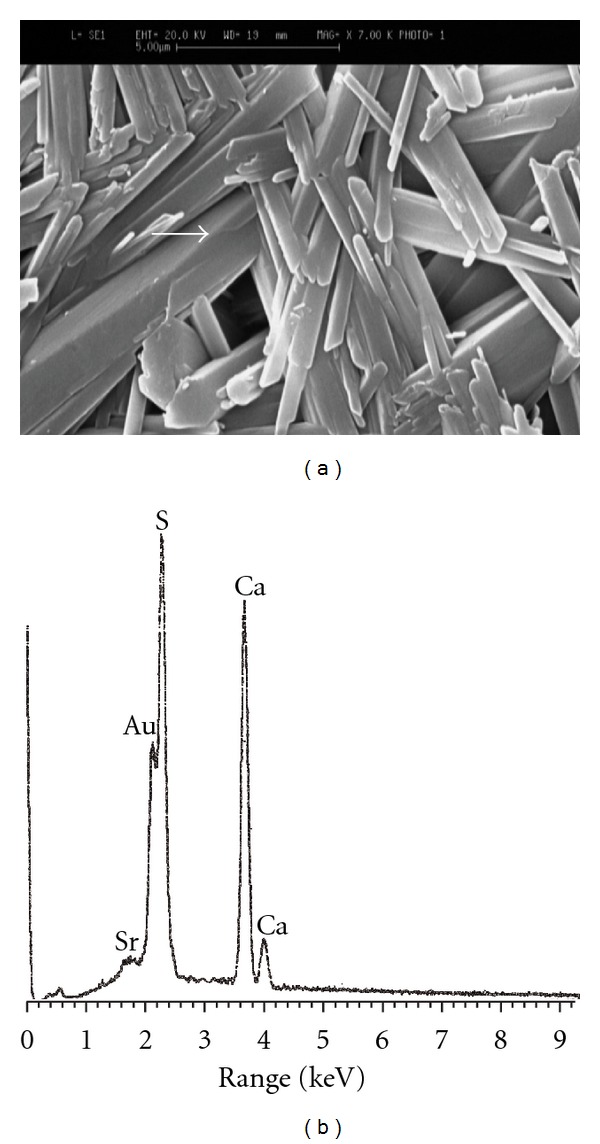
The SEM image of G-Sr1 specimen (a) along with its corresponding EDXA patterns (b).

**Figure 5:**
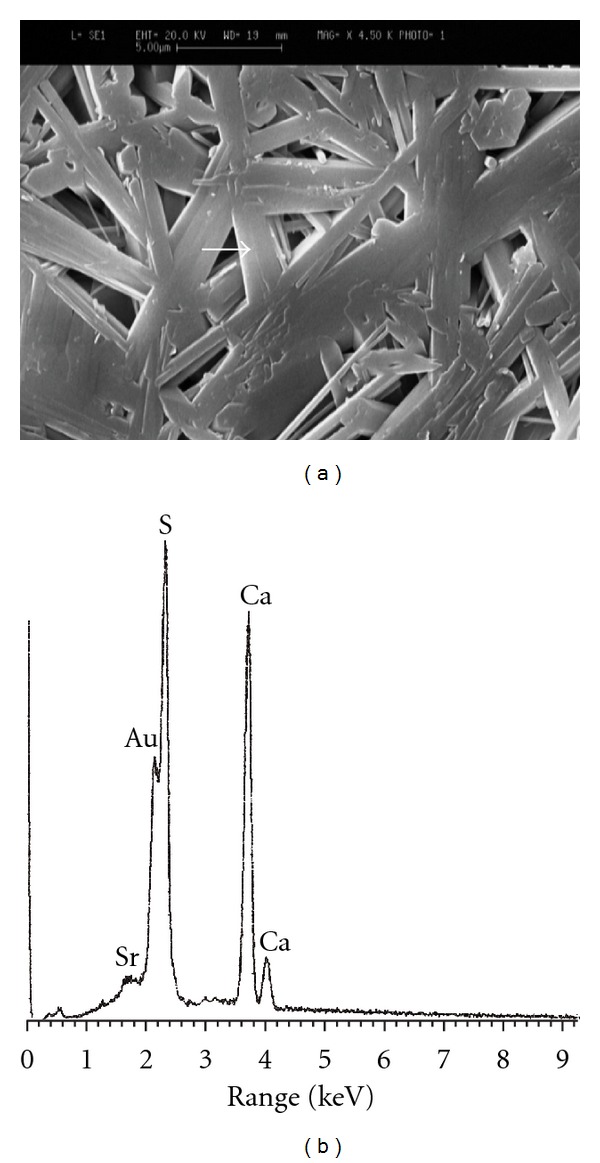
The SEM image of G-Sr2 specimen (a) along with its corresponding EDXA patterns (b).

**Figure 6:**
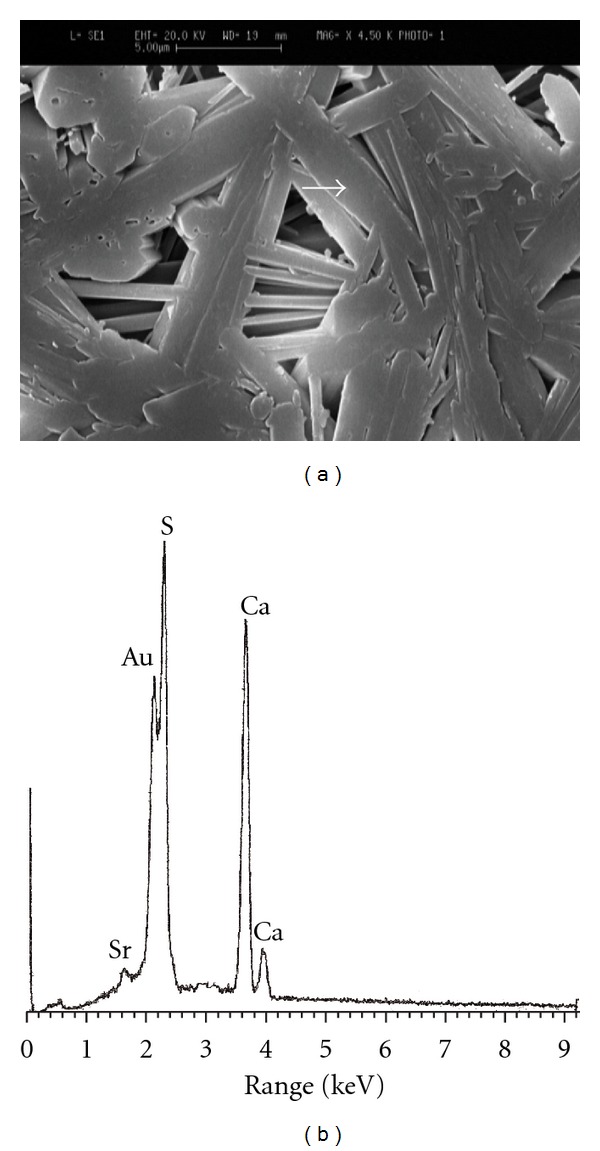
The SEM image of G-Sr4 specimen (a) along with its corresponding EDXA patterns (b).

The SEM images of the specimens along with their corresponding EDXA patterns are illustrated in Figures 3 to 6. Microstructure of pure gypsum (Figure 3(a)) was found to consists of many rod-like crystals entangled to each other and its corresponding EDXA pattern (Figure 3(b)) shows the presence of calcium and sulfur as main elements of gypsum as well as gold (Au) element created from the coating layer. Micrographs of Sr-doped gypsum specimens (Figure 4: G-Sr1, Figure 5: G-Sr2 and Figure 6: G-Sr4) show thicker rod-like crystals with more elongated and compacted morphologies compared to pure gypsum. In these images, the EDXA patterns taken from that crystal marked with an arrow reveal the presence of strontium dopant element in the composition of gypsum. Regarding uniform morphology of the crystals and lack of other phases in the gypsum:Sr (from XRD data), these EDXA patterns can be used as complementary proofs for incorporation of Sr ions into lattice structure of gypsum.

### 3.5. Density

**Figure 7:**
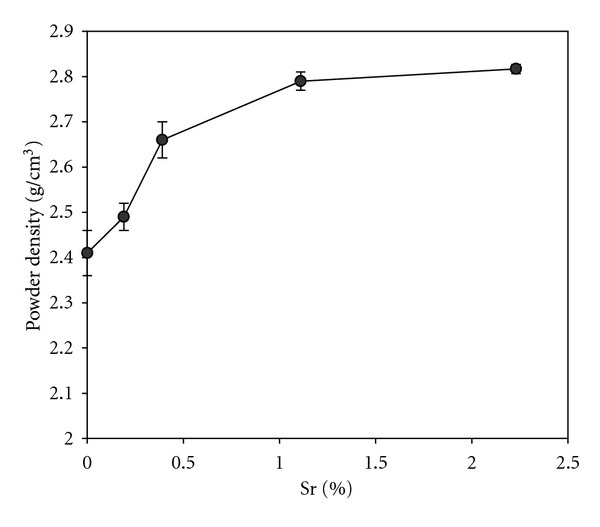
The powder density of gypsum specimens containing different contents of Sr dopant.

Figure 7 shows the effect of incorporation of Sr^2+^ ions into gypsum on its powder density. An increase in density of gypsum powder is observed by adding Sr in which the increase correlates with Sr concentration and the differences are statistically significant (*P* < 0.05). The increased powder density of Sr-doped gypsum in comparison with pure gypsum is due to the higher atomic weight of Sr (87.6 g/mol) compared to Ca (40.0 g/mol).

### 3.6. Compressive Strength

**Figure 8:**
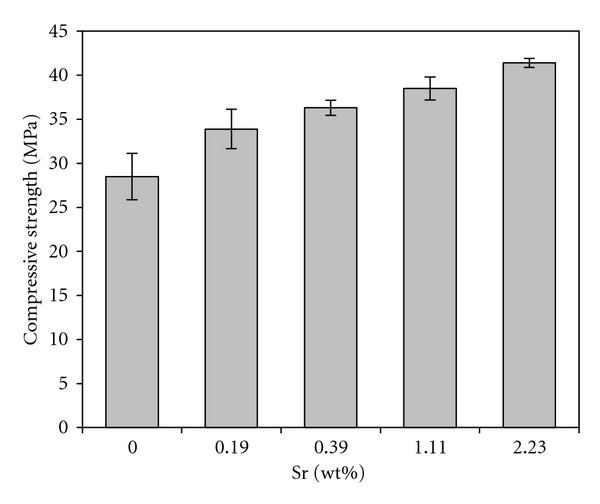
Compressive strength of gypsum specimens containing different contents of Sr dopant.

Figure 8 shows the compressive strength of the gypsum bioceramics doped with various contents of strontium. There is no significant difference between the compressive strength values of the gypsum containing various amounts of Sr (*P* > 0.05). However the compressive strength value of pure gypsum is significantly lower than that of Sr-doped ones. Generally, mechanical strength of ceramic bodies is ruled by their microstructural features. In the case of calcium sulfate, entanglement of rod-like crystals is responsible for mechanical hardening phenomenon. These crystals are gypsum produced from conversion of calcium sulfate hemi-hydrate to calcium sulfate dihydrate through a dissolution-precipitation process [25]. The size (length and thickness) of these entangled crystals and their compression in the microstructure can affect the mechanical properties of gypsum. Note that thick and protracted crystals of gypsum:Sr tightly locked to each other (Figures 4 to 6) were to find why the compressive strength of pure gypsum is lower than that of gypsum:Sr.

### 3.7. Solubility and Strontium Release

**Figure 9:**
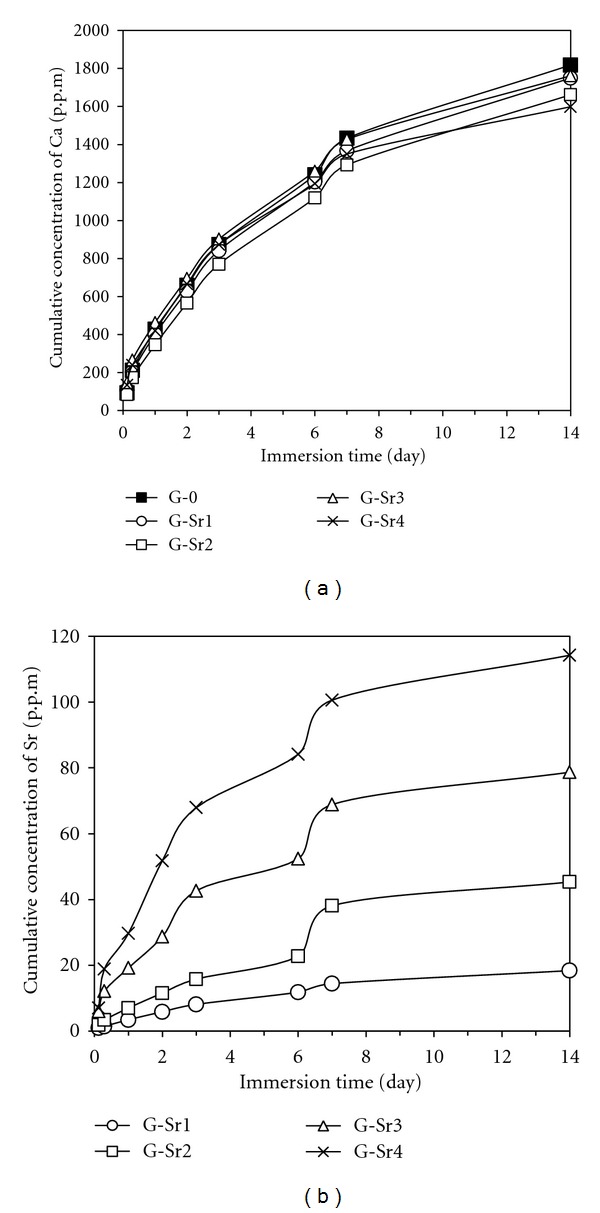
Cumulative concentration of Ca (a) and Sr (b) ions released from various Sr-doped specimens into the SBF solution.

The ion release of gypsum ceramics in the SBF solution is shown in Figure 9. This experiment was carried out with periodic exchange of SBF solution and the results are presented as cumulative concentration of Ca (Figure 9(a)) and Sr (Figure 9(b)) against the immersion time. According to Figure 9(a), the concentration of Ca ions released from all gypsum specimens is much higher than that of other calcium phosphates reported in other studies [26]. It originates from the higher solubility and thus higher bioresorption rate of calcium sulfate-based materials in comparison with other well-known bioceramics such as *β*-tricalcium phosphate and hydroxyapatite. The concentration of Ca^2+^ ions released from pure gypsum (G-0) into the SBF solution was slightly higher than that of gypsum:Sr specimens. Since the calcium concentration is proportional to resorption rate of such bioceramics, the results demonstrate that incorporation of strontium ions into gypsum can diminish the biomaterial resorption rate. This decreased degradation rate of gypsum:Sr indicates higher chemical stability of these materials compared to pure gypsum. It is suggested that this is due to the complicated movement Ca ions in the crystal when encountering with Sr ions having larger atomic radius. The higher bonding strength of Sr-sulfate group in comparison with Ca-sulfate group should not be ignored too (note that Sr is more electronegative than Ca [27]). Calcium sulfate-based bioceramics have been used as potential materials for bone tissue regeneration, because of their adequate biocompatibility and osteoconductivity. However, the high degradation rate of these materials is the main practical problem [28], resulting in material to be lost in defect site before the completion of tissue repair. Incorporation of strontium into gypsum may control its too fast resorption rate to some extent.

The results clearly showed a continuous release of incorporated strontium ions into the SBF medium. It means that the gypsum can act as a suitable material for releasing Sr and stimulating osteoblastic cells and tissue repair. The concentration of Sr^2+^ ions in the SBF is proportional to the Sr content in the gypsum. At the end of the evaluation period (14 day), cumulative Sr release was 18 mg/g/L for G-Sr1 (about 4.7–8.9 mmol/g/day) and 114 mg/g/L for G-Sr4 (about 20–25 mmol/g/day). Regarding the therapeutic dose of Sr^2+^ (as strontium ranelate) which is in the range of 2.4–8.75 mmol/day (per kg of rat) for oral administration [4], the level of Sr^2+^ ions released from gypsum:Sr of this study is higher than that of Sr-substituted hydroxyapatite reported by Landi et al. [29] and comparable to that of Sr-doped beta-tricalcium phosphate bioceramics reported by Alkhraisat et al. [26]. The concentration of the released Sr^2+^ ions should be in therapeutic range, because osteoclasts are inhibited by the effective dose of these ions. The maximum Sr/Ca molar ratio in the SBF is about 0.03, which is in the range of Sr/Ca in bone skeleton [24]. Generally, our results showed that the release of Sr^2+^ ions into an SBF and thus into a cell culture medium can be tailored by varying its concentration in gypsum crystals.

### 3.8. Cell Proliferation and Alkaline Phosphatase Activity

**Figure 10:**
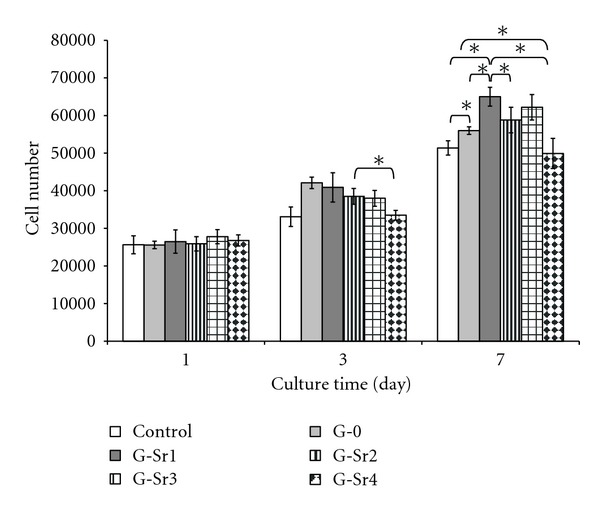
Proliferation of G-292 osteoblastic cells on gypsum specimens with various amounts of Sr dopant (*P < 0.05).

Figure 10 shows the results of G-292 cell proliferation on the polystyrene (control), pure gypsum, and Sr-doped gypsum specimens. After cell attachment, the osteoblasts proliferate on all specimens because of the difference in cell number between days 1, 3 and 7 (*P* < 0.05). In this study, it was found that the proliferation of the cells on gypsum specimens was significantly (*P* < 0.05) better than on control specimen, meanwhile the osteoblastic cells could proliferate on some gypsum:Sr discs at a significantly higher rate than on pure gypsum. Firstly, according to Lazáry et al. [21], physical structure of gypsum may be responsible for the suitable proliferation of osteoblastic cells onto the gypsum specimen. In other words, the cells can be attached onto the gypsum surfaces because it has been formed from Plaster of Paris (CaSO_4_-hemihydrate) by adding water and its microstructure is composed of too many rod-like small crystals entangled to each other to provide large specific surface area. Secondly, it has been proved that strontium ions can stimulate cellular responses and subsequently increase rate of cell proliferation process. In this study, the effect of Sr^2+^ on proliferation of osteoblastic cells was proved to be dose-dependent, because of the lower proliferation rate of the osteoblasts on G-Sr4 compared with other Sr-doped samples. It is in agreement with other studies [30].

**Figure 11:**
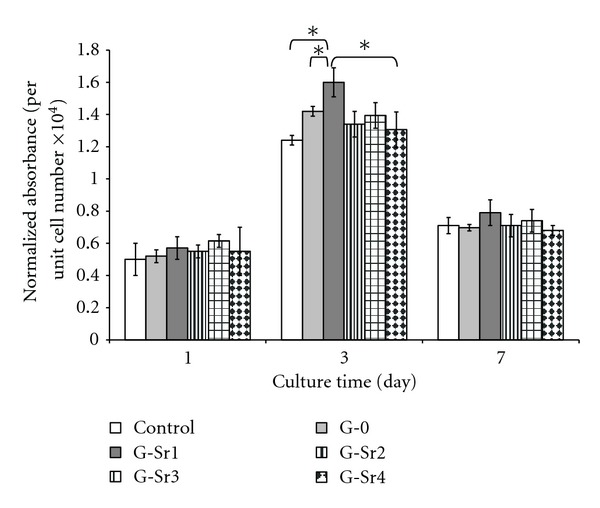
Normalized ALP activity of G-292 osteoblastic cells cultured on gypsum specimens with various amounts of Sr dopant (*P < 0.05).

Alkaline phosphatase is known as an early osteoblastic differentiation marker and is produced by the cells showing mineralized extracellular matrix [31]. Alkaline phosphatase activity of the osteoblasts is illustrated in Figure 11 as normalized absorbance number (per unit cell number) against the culture time. ALP activity of the osteoblasts cultured on gypsum specimens, especially G-Sr1, was better than those cultured on polystyrene. While higher ALP activity was found on G-Sr1 compared to gypsum, the difference in ALP value of pure gypsum and other Sr-doped gypsum specimens was not statistically significant (*P* > 0.05). Alkaline phosphatase activity was inhibited on day 7 for all specimens probably due to conflux of extended cell [16]. There are many studies that confirm this fact that strontium ions incorporated into composition of bioceramics can improve proliferation and/or alkaline phosphatase activity of osteoblastic cells [32]. In this study, it was shown that the presence of Sr^2+^ ions in the lattice structure of gypsum could also play important role in proliferation and ALP activity of G-292 osteoblastic cells.

## 4. Conclusions

Calcium sulfate dihydrate (gypsum) bioceramics with improved compressive strength and cellular properties are obtained by doping various concentrations of strontium into crystal lattice of the material. Incorporation of strontium into gypsum affects its crystal morphology and water solubility. The Sr-doped gypsum was found to be useful as strontium releasing material with a continuous release profile in which amount of the liberated strontium is dependent of its concentration in host material. Both proliferation rate and alkaline phosphatase activity of human osteosarcoma cells on gypsum:Sr can be improved by incorporation of strontium, depending on the Sr dose in the host lattice. These observations can also predict better in vivo functions of strontium-doped gypsum compared to pure gypsum which requires more investigations.

## Figures and Tables

**Table 1:** Details of various paste formulations for preparation of Sr-doped gypsum along with their names.

	Name of specimen				
	G-0	G-Sr1	G-Sr2	G-Sr3	G-Sr4
Assumed Sr content in gypsum (%)	0	0.25	0.5	1.25	2.5
Concentration of Sr(NO_3_) solution used as liquid phase in paste (%)	0	1.36	2.80	7.04	14.08
Solid phase of paste	*α*-CHS	*α*-CHS	*α*-CHS	*α*-CHS	*α*-CHS

**Table 2:** The weight percentage of Ca and Sr elements in various Sr-doped gypsum specimens (measured by ICP-AES technique).

	G-0	G-Sr1	G-Sr2	G-Sr3	G-Sr4
Ca (%)	22.11	21.79	21.65	20.18	19.79
Sr (%)	<10 p.p.m	0.19	0.38	1.11	2.23

## References

[1] Dahl SG, Allain P, Marie PJ (2001). Incorporation and distribution of strontium in bone. *Bone*.

[2] Pors Nielsen S (2004). The biological role of strontium. *Bone*.

[3] Rizzoli R (2005). A new treatment for post-menopausal osteoporosis: strontium ranelate. *Journal of Endocrinological Investigation*.

[4] Marie PJ, Ammann P, Boivin G, Rey C (2001). Mechanisms of action and therapeutic potential of strontium in bone. *Calcified Tissue International*.

[5] Suzuki O, Imaizumi H, Kamakura S, Katagiri T (2008). Bone regeneration by synthetic octacalcium phosphate and its role in biological mineralization. *Current Medicinal Chemistry*.

[6] Huan Z, Chang J (2009). Calcium-phosphate-silicate composite bone cement: self-setting properties and in vitro bioactivity. *Journal of Materials Science*.

[7] Yu T, Ye J, Wang Y (2009). Synthesis and property of a novel calcium phosphate cement. *Journal of Biomedical Materials Research—Part B*.

[8] Bigi A, Foresti E, Gandolfi M, Gazzano M, Roveri N (1997). Isomorphous substitutions in *β*-tricalcium phosphate: the different effects of zinc and strontium. *Journal of Inorganic Biochemistry*.

[9] Jegou Saint-Jean S, Camiré CL, Nevsten P, Hansen S, Ginebra MP (2005). Study of the reactivity and in vitro bioactivity of Sr-substituted *α*-TCP cements. *Journal of Materials Science*.

[10] Li YW, Leong JCY, Lu WW (2000). A novel injectable bioactive bone cement for spinal surgery: a developmental and preclinical study. *Journal of Biomedical Materials Research*.

[11] Guo D, Xu K, Zhao X, Han Y (2005). Development of a strontium-containing hydroxyapatite bone cement. *Biomaterials*.

[12] Cheung KMC, Lu WW, Luk KDK (2005). Vertebroplasty by use of a strontium-containing bioactive bone cement. *Spine*.

[13] Wong CT, Lu WW, Chan WK (2004). In vivo cancellous bone remodeling on a Strontium-containing hydroxyapatite (Sr-HA) bioactive cement. *Journal of Biomedical Materials Research—Part A*.

[14] Xue W, Hosick HL, Bandyopadhyay A (2007). Preparation and cell-materials interactions of plasma sprayed strontium-containing hydroxyapatite coating. *Surface and Coatings Technology*.

[15] Abou Neel EA, Chrzanowski W, Pickup DM (2009). Structure and properties of strontium-doped phosphate-based glasses. *Journal of the Royal Society Interface*.

[16] Hesaraki S, Alizadeh M, Nazarian H, Sharifi D (2010). Physico-chemical and in vitro biological evaluation of strontium/calcium silicophosphate glass. *Journal of Materials Science*.

[17] Anson D (2000). Using calcium sulfate in guided tissue regeneration: a recipe for success. *Compendium of Continuing Education in Dentistry*.

[18] Bier SJ, Sinensky MC (1999). The versatility of calcium sulfate: resolving periodontal challenges. *Compendium of Continuing Education in Dentistry*.

[19] Pecora G, Andreana S, Margarone JE, Covani U, Sottosanti JS (1997). Bone regeneration with a calcium sulfate barrier. *Oral Surgery, Oral Medicine, Oral Pathology, Oral Radiology, and Endodontics*.

[20] Paderni S, Terzi S, Amendola L (2009). Major bone defect treatment with an osteoconductive bone substitute. *La Chirurgia Degli Organi di Movimento*.

[21] Lazáry A, Balla B, Kósa JP (2007). Effect of gypsum on proliferation and differentiation of MC3T3-E1 mouse osteoblastic cells. *Biomaterials*.

[22] Kokubo T, Kushitani H, Sakka S, Kitsugi T, Yamamuro T (1990). Solutions able to reproduce in vivo surface-structure changes in bioactive glass-ceramic A-W3. *Journal of Biomedical Materials Research*.

[23] Hesaraki S, Moztarzadeh F, Nezafati N (2009). Evaluation of a bioceramic-based nanocomposite material for controlled delivery of a non-steroidal anti-inflammatory drug. *Medical Engineering and Physics*.

[24] Fourman P, Royer P, Levell M, Morgan DB (1968). *Calcium Metabolism and the Bone*.

[25] Hesaraki S, Moztarzadeh F, Nemati R, Nezafati N (2009). Preparation and characterization of calcium sulfate-biomimetic apatite nanocomposites for controlled release of antibiotics. *Journal of Biomedical Materials Research—Part B*.

[26] Hamdan Alkhraisat M, Moseke C, Blanco L, Barralet JE, Lopez-Carbacos E, Gbureck U (2008). Strontium modified biocements with zero order release kinetics. *Biomaterials*.

[27] Boyd D, Towler MR, Watts S, Hill RG, Wren AW, Clarkin OM (2008). The role of Sr^2+^ on the structure and reactivity of SrO-CaO-ZnO-SiO_2_ ionomer glasses. *Journal of Materials Science*.

[28] Rauschmann MA, Wichelhaus TA, Stirnal V (2005). Nanocrystalline hydroxyapatite and calcium sulphate as biodegradable composite carrier material for local delivery of antibiotics in bone infections. *Biomaterials*.

[29] Landi E, Tampieri A, Celotti G, Sprio S, Sandri M, Logroscino G (2007). Sr-substituted hydroxyapatites for osteoporotic bone replacement. *Acta Biomaterialia*.

[30] Xue W, Moore JL, Hosick HL (2006). Osteoprecursor cell response to strontium-containing hydorxyapatite ceramics. *Journal of Biomedical Materials Research—Part A*.

[31] Stein GS, Lian JB, Owen TA (1990). Relationship of cell growth to the regulation of tissue-specific gene expression during osteoblast differentiation. *The FASEB Journal*.

[32] Kim HW, Koh YH, Kong YM, Kang JG, Kim HE (2004). Strontium substituted calcium phosphate biphasic ceramics obtained by a powder precipitation method. *Journal of Materials Science*.

